# Landmark and route knowledge in children’s spatial representation of a virtual environment

**DOI:** 10.3389/fpsyg.2014.01522

**Published:** 2015-01-23

**Authors:** Marion Nys, Valérie Gyselinck, Eric Orriols, Maya Hickmann

**Affiliations:** ^1^INSERM UMR S894 and Laboratoire Mémoire et Cognition, Institut de Psychologie, Université Paris Descartes – Sorbonne Paris-CitéParis, France; ^2^INSERM UMR S894, Centre de Psychiatrie et NeurosciencesParis, France; ^3^CNRS, Laboratoire Structures Formelles du Langage, UMR 7023, Université de Paris 8Paris, France

**Keywords:** cognitive development, decisional landmark, individual differences, language abilities, verbal encoding, visuo-spatial encoding, virtual reality, wayfinding

## Abstract

This study investigates the development of landmark and route knowledge in complex wayfinding situations. It focuses on how children (aged 6, 8, and 10 years) and young adults (*n* = 79) indicate, recognize, and bind landmarks and directions in both verbal and visuo-spatial tasks after learning a virtual route. Performance in these tasks is also related to general verbal and visuo-spatial abilities as assessed by independent standardized tests (attention, working memory, perception of direction, production and comprehension of spatial terms, sentences and stories). The results first show that the quantity and quality of landmarks and directions produced and recognized by participants in both verbal and visuo-spatial tasks increased with age. In addition, an increase with age was observed in participants’ selection of decisional landmarks (i.e., landmarks associated with a change of direction), as well as in their capacity to bind landmarks and directions. Our results support the view that children first acquire landmark knowledge, then route knowledge, as shown by their late developing ability to bind knowledge of directions and landmarks. Overall, the quality of verbal and visuo-spatial information in participants’ spatial representations was found to vary mostly with their visuo-spatial abilities (attention and perception of directions) and not with their verbal abilities. Interestingly, however, when asked to recognize landmarks encountered during the route, participants show an increasing bias with age toward choosing a related landmark of the same category, regardless of its visual characteristics, i.e., they incorrectly choose the picture of another fountain. The discussion highlights the need for further studies to determine more precisely the role of verbal and visuo-spatial knowledge and the nature of how children learn to represent and memorize routes.

## INTRODUCTION

The spatial representation of large-scale environments is often studied within the theoretical framework of mental models (Johnson-Laird, 1983). Mental models can be constructed from perception, imagination, or discourse, and can therefore imply both verbal and visuo-spatial modes of encoding information in our spatial representation. Constructing such a representation is necessary for wayfinding, which is a complex process involving the ability to learn, to recall, and to follow a route through the environment (Blades, 1991). In order to find our way, we use all sorts of spatial knowledge, including landmark knowledge, route or procedural knowledge, and survey knowledge (Siegel and White, 1975; Golledge, 1987). A landmark is a visual entity that might play a role in our wayfinding activity, and that is perceived and remembered on the basis of its properties, such as its shape and structure (Golledge et al., 1985; Presson and Montello, 1988), its sociocultural significance or symbolic function (Lynch, 1960; Appleyard, 1969). Route knowledge includes various types of static and dynamic information, such as the sequential order of landmarks encountered and of the decisions taken, as well as associations between landmarks and changes in direction (Buchner and Jansen-Osmann, 2008; Farran et al., 2012). Survey knowledge corresponds to an overview of the environment, for example as represented by a map showing spatial relationships between routes and landmarks, enabling us to find shortcuts.

A number of questions have arisen in previous studies of the development of spatial knowledge, concerning for example the nature of multiple abilities that might be necessary for children to construct efficient representations of their environment or the timing of development from infancy to later childhood and to adulthood. As discussed below, the present study addresses some of these questions by focusing on the development of landmark and route knowledge using a virtual environment in children aged 6–10 years and in young adults. Furthermore, the study explores the extent to which verbal vs. visuo-spatial encoding of information has an impact on how children and adults construct spatial models, as well as whether general verbal and non-verbal abilities play a role in this process.

Developmental studies diverge with respect to how early they report spatial abilities to be available to children. Depending on the studies and on the abilities examined, divergent results range from reports claiming that some spatial abilities are available from birth on to claims that they are not mastered until much later during childhood or even adulthood. Thus, according to some authors (Spelke et al., 1992; Spelke, 1998), spatial cognition seems to partially provide infants with innate “core” knowledge necessary to process spatial information, although many of the skills involved still need some time to further develop and to become fully mature (Vasilyeva and Lourenco, 2012, for review). For example, object permanence is observed in infants as early as 2 or 3 months of age (Baillargeon, 1987; Baillargeon and DeVos, 1991), although it had been previously predicted (e.g., by Piagetian theory) to develop at much later stages (after one year of age). In addition, discrimination between different spatial relations such as ABOVE vs. BELOW can be observed at 6 months (Quinn, 1998), despite the fact that children do not acquire the corresponding spatial prepositions until later in development (Johnston, 1988). Other strikingly early skills have been reported, such as memory for the location of one element at 6 months and for multiple locations (up to three objects in different places) at 8–9 months (Oakes et al., 2011; Richmond et al., 2015). Among abilities that develop slightly later, one finds an improvement in spatial memory (assessed by a search task for hidden objects) between 2 and 3½ years (Ribordy et al., 2013), followed by later developments between 3 and 10 years (e.g., Lehnung et al., 1998; Nardini et al., 2006). These examples of early vs. later cognitive abilities raise some methodological questions, such as how to compare findings that are based on very diverse tasks assessing spatial knowledge, but they have opened fundamental debates concerning the innate vs. acquired nature of spatial knowledge. It is worth keeping these debates in mind, although they are beyond the scope of our paper, which tested children between the ages of 6–10 years. In the following, we therefore focus mostly on developments that have been observed during later phases.

Siegel and White (1975) postulate that the development of spatial knowledge during childhood evolves from landmark to route knowledge and then from route knowledge to survey knowledge. At each step, new abilities are necessary. Siegel and White’s (1975) proposal was tested in a study by Cousins et al. (1983) evaluating children’s spatial knowledge of their school campus at 7, 10, and 13 years. All children performed well on wayfinding, most on landmark knowledge, fewer on route ordering, fewer still on route scaling, and only very few performed well on a configuration task (estimating the positions of landmarks). Performance in most of these tasks increased with age but with some variability in children across the tasks. A number of other studies report early abilities to use survey knowledge (e.g., Hazen et al., 1978; Huttenlocher et al., 2008). For example, Huttenlocher et al. (2008) asked children between 3 and 4 years to use a scale model in order either to place or to find an object in particular place space. Children were able to place an object at the correct place as early as 3½ years and to find an object at 4 years. This early ability to use survey knowledge contradicts Siegel and White’s (1975) proposal of a gradual development of spatial knowledge from landmark to survey knowledge.

The fact that spatial knowledge observed at preschool age is followed by further gradual developments at later ages has been reported in many studies. Some show that young children are able to handle landmark knowledge but that their performance nonetheless improves later on. Children as young as 5 years of age can use landmarks for wayfinding (e.g., Fenner et al., 2000; Jansen-Osmann et al., 2007; Bullens et al., 2010) or to find a hidden object (e.g., Nardini et al., 2006). These studies also show that they must first learn how to pay attention to them, suggesting that attention is a general ability that is important in such tasks. Cornell et al. (1989) show that advising 6- and-12-year-old children to pay attention to proximal landmarks (i.e., landmarks near the route) help them retrace a route successfully. By contrast, distant landmarks (i.e., landmarks seen from far away) help older children (12 years), but not younger ones, find their way when they are placed off the correct route. This suggests that younger children only rely on proximal landmarks, whereas older children can also use distant landmarks, which provide a more global point of view. In a second study (Heth et al., 1997) both 8- and 12-year-old children were able to report four objects designated as landmarks (e.g., a bike). They were also able to use other additional landmarks (not previously indicated), but older children mentioned overall more landmarks, including more stable and distant landmarks, in addition to the designated landmarks that helped them find correct directions.

Concerning landmark knowledge, further studies help elucidate the various conditions under which spatial knowledge can be used and the various factors that may influence children’s ability to rely on this knowledge efficiently when solving wayfinding tasks. Some studies show the influence of the positions of landmarks in the development of children’s spatial representations. Cohen and Schuepfer (1980) asked children aged 7–8 and 11–12 years as well as adults to learn a route in a maze by presenting slides containing landmarks with different functions (adjacent to correct vs. incorrect wayfinding decisions or in the absence of any such decisions). Landmarks that were adjacent to correct wayfinding decisions (i.e., decisional landmarks) were significantly better remembered than others. However, younger children recalled fewer landmarks than older children and adults. Jansen-Osmann and Wiedenbauer (2004) replicated this study with virtual navigation in the same maze using toy animals as landmarks. Overall, these authors obtained the same results except that, contrary to Cohen and Schuepfer (1980), they mostly showed no difference between age 11–12 years and adults. This finding suggests that a virtual presentation, closer to a real navigation, may help children integrate the path more easily.

The studies presented so far show that as early as 7 years of age, children are able to select strategic landmarks. Younger children are less prolific and precise during recall than older children. During simple navigation in a maze older children are able to find their way as well as adults, whereas younger children have more difficulties. All groups are able to use landmarks but wayfinding performance changes with age. Thus, contrary to young children who base their strategy almost exclusively on landmarks, particularly on proximal ones, older children and adults also use other strategies that allow them to find their way.

Some other studies indicate that the way in which information is organized may influence landmark knowledge. Jansen-Osmann and Fuchs (2006) asked children aged 7–8 and 11–12 years as well as adults to explore a virtual maze (without any landmark, with landmarks that were randomly placed, or with landmarks placed by category, i.e., types of fruit at changing points and of animals at non-changing points). The presence of landmarks facilitated wayfinding performance (number of trials needed to reach the goal) of both children and adults, but the beneficial effect of landmarks was more important for children at 7–8 years. In addition, the presence of landmarks had no influence on reorientation behavior (turns back to the starting point, 180° turnarounds, segments followed twice), nor on survey knowledge (estimating direction, finding the shortest detour and the position of targets on a map). Finally, in comparison to random placement, the presence of landmarks placed according to their category improves location knowledge. Hund and Plumert (2003) asked children aged 7, 9, 11 years and adults to learn the location of objects that belonged to four categories (animals, vehicles, food, and clothes). Same-category members were either all located in the same quadrant of a square box, or objects and locations were randomly paired. Results show that categorical features of landmarks within the same class (e.g., food) negatively affected memory of their disposition: categorically related landmarks were placed closer to the center of the quadrant than unrelated ones. This categorical bias in location memory decreased with increasing age and could be due to children’s difficulty in differentiating elements that belong to the same category. If we assume that knowledge of categories such as those above depends at least partially on verbal knowledge (e.g., information encoded in the lexicon), then the findings of this study may indicate the existence of potential language effects on our knowledge of spatial configurations. Language is indeed often viewed as influencing how we construct and modulate our representations, including in the spatial domain (Levinson et al., 2002; Shusterman et al., 2011). It is therefore of interest to determine whether linguistic abilities, such as knowledge of labels that may partially underlie categorization, have an influence on the visual recognition of landmarks.

Individual differences in spatial knowledge have been observed with both children and adults but have been often neglected (Newcombe et al., 2013). These differences have been mostly linked to cognitive abilities (e.g., Quaiser-Pohl et al., 2004; Fields and Shelton, 2006). More recently, Purser et al. (2012) also observed that the increasing ability to learn a virtual maze between 5 to 12 years was linked to general cognitive abilities (vocabulary, working memory, executive functions, and episodic memory). It further seems that verbal and visuo-spatial abilities both have a specific influence on spatial representations. Very few studies, however, have investigated the impact of both types of abilities on the development of spatial representations. A study (Fenner et al., 2000) indicates that they do not seem to play the same role in a navigation task during which the experimenter walked with children aged 5–6 and 9–10 years in a college. In addition to the navigation task, various cognitive abilities considered by the authors as visuo-spatial (mental rotation, spatial span, reasoning) and verbal (knowledge of vowels, word endings, comprehension, digit span) were measured by brief tasks. Analyzes showed an asymmetrical effect of visuo-spatial and verbal abilities on the development of wayfinding abilities. Children with high visuo-spatial abilities had superior wayfinding abilities than those with low visual-spatial abilities, but no influence of verbal abilities was found.

To summarize, adults and children both use landmarks to orient themselves in a new environment and to help find their way. However, children must learn to pay attention to landmarks and to relevant information when selecting them. In addition, whereas younger children mainly use landmarks to orient themselves, older children become able to use other strategies. Some studies suggest that categorization enhances the number of landmarks remembered although it can also result in a lack of distinctiveness that creates some difficulties for children when they have to accurately position these entities. Among the many tasks that can be used to assess spatial knowledge, it is important to select a number of verbal and visuo-spatial tasks in such a way as to be able to assess the role of multiple types of encoding. Some studies have shown progressions in landmark and/or route knowledge using tasks that require either verbal or non-verbal knowledge. In this respect, as shown below, the way in which we study representations can determine what we can learn about them. Such differences between verbal and non-verbal knowledge have been indeed observed in adults (see e.g., Gras et al., 2013; Picucci et al., 2013). They should also be observed in children whose visuo-spatial and verbal abilities are developing at the same time. However, note that these abilities may develop at different rhythms, which might produce more discrepancy in performance in these two types of tasks. For a given stimulus, children’s verbal and non-verbal performance may vary with age, i.e., performance in one type of task may become easier, more difficult, or equivalent to performance in the other as a function of developmental period. Given prior research showing very early spatial abilities after only a few months of life (Oakes et al., 2011; Ribordy et al., 2013; Richmond et al., 2015), it may be expected that language abilities may develop later than non-verbal abilities. For example, whereas very young children can understand a simple spatial relation enabling them to find a toy, the ability to refer precisely to this relation by means of language develops later and enriches prior knowledge which becomes more precise. Another possibility, however, is that both verbal and non-verbal abilities develop together and so no differences should be expected in preferences for one or the other type of encoding at any age.

Furthermore, little is still known about how verbal and non-verbal knowledge interact with each other during the development of children’s spatial representations, as well as how they interact with the development of more general abilities. The present study aims to fill these gaps by measuring verbal and visuo-spatial knowledge of landmarks and directions in production and recognition tasks on the basis of a complex virtual environment. We chose to study the development of spatial representation based on virtual reality with desktop systems (virtual environment on a projection screen). Although this methodology may raise some questions, for example concerning the extent to which virtual reality can approximate the real world, it has major advantages for this type of research, for example providing: easy, economic, and systematic ways of varying and controlling environmental features (e.g., manipulating the structure of the town, the landmarks …); an ecological and playful method to investigate the development of children’s spatial knowledge and behavior (Jansen-Osmann, 2007). We also investigate below whether similar results are found in a virtual town comprising many landmarks as well as non-specific information (buildings, street furniture …) in comparison to studies using a maze with landmarks but without any non-specific information (e.g., Jansen-Osmann et al., 2007).

Two main questions were considered in this study: (1) the development of spatial knowledge and (2) the extent to which individual differences in general cognitive abilities have an impact on spatial knowledge.

First, concerning the development of spatial knowledge, both verbal and visuo-spatial assessments were expected to show an early increase in landmark knowledge (quantity and quality of landmarks remembered and produced) followed by a later increase in route knowledge (memory for directions and knowledge of the links between landmarks and directions). Young children should acquire and use mostly general landmark knowledge, but not route knowledge which requires more global organizational capacities. They should be able to mention only few landmarks and fewer directions, and they should not be able to indicate these entities on a general representation like a map. In addition, they should not be much able to bind both types of information. By contrast, older children should be more precise in their landmark knowledge (mentions and placements of landmarks) and acquire more complete route knowledge than younger ones, displaying a representation that binds together multiple types of information. Links between landmarks and directions should therefore increase, especially for decisional landmarks associated to changes of direction. We also examined possible differences that may occur in participants’ preferred form of encoding when constructing spatial representations as a function of age. Three possibilities were explored. A first possibility is that young children should find it easier to base their representation mostly on a visuo-spatial encoding (e.g., pictures of landmarks) given that visual abilities are observed before verbal ones and that language skills take some time to develop (e.g., spatial prepositions), while older children and adults should be able to rely on both verbal and visuo-spatial encoding (e.g., pictures and labels of landmarks). A second possibility is that, given the age range examined here (from 6 years on), language may have already sufficiently developed to provide a useful symbolic system that may enhance performance in various tasks (e.g., by providing retrieval cues that improve memory). A third possibility is that both verbal and non-verbal abilities develop together and so no differences should be expected between the various tasks formats.

Second, concerning individual differences, although different tests involving large-scale spatial representations should be related to each other indicating a general spatial understanding of the environment, it was also expected that they should be differentially related to either visuo-spatial or verbal abilities. Whereas visuo-spatial abilities should be strongly linked to good landmark and route performance (particularly when measures of performance are non-verbal), verbal abilities should be mostly linked to verbal production as well as to the ability to bind and organize information. In addition, as previously shown with virtual environments, attention was expected to be related to all spatial representation measures, including visuo-spatial and verbal ones.

## MATERIALS AND METHODS

### PARTICIPANTS

Sixty-two healthy children at three grade levels (kindergarten, second- and fourth-graders) and 30 young adults participated in the study. Three children and ten adults were excluded because they were bilingual (six adults, one child), color blind (two adults), dyslexic (one adult), or had an abnormally low attention performance (two children, one adult). The resulting sample included: 22 preschoolers (13 girls and 9 boys, mean age 6;3 years, range 4 months), 18 second-graders (9 girls and 9 boys, mean age 8;7 years, range 5 months), 19 fourth-graders (9 girls and 10 boys, mean age 10;1 years, range 5 months) and 20 adults (11 women and 9 men, mean age 21 years, range 15 months). Children were recruited through advertisements at schools, with signed consent of school directors, teachers, and parents. The adults were recruited through advertisement at the university and signed a consent form.

### MATERIAL

A questionnaire evaluated participants’ hand lateralization, their use of computers (how often they worked or played with computer or other virtual games such as “Wii”), the frequency with which they walked alone (in usual or new environments), and their linguistic history (native language, frequency, and destination of travel abroad …). An ANOVA shows a significant increase with age in the habit to walk alone [*F*(3,71) = 70.25, *p* < 0.0001, η^2^= 0.75] and to use computers or video games [*F*(3,71) = 22.87, *p* < 0.0001, η^2^= 0.75]. No difference was found among children of 6 and 8 years with respect to their use of computers (from a few times a year to a few times every week) but the use of computers increased between 8 and 10 years (*p* < 0.05) and between 8 years and adults (*p* < 0.0001).

#### Virtual route and tasks

**FIGURE 1:**
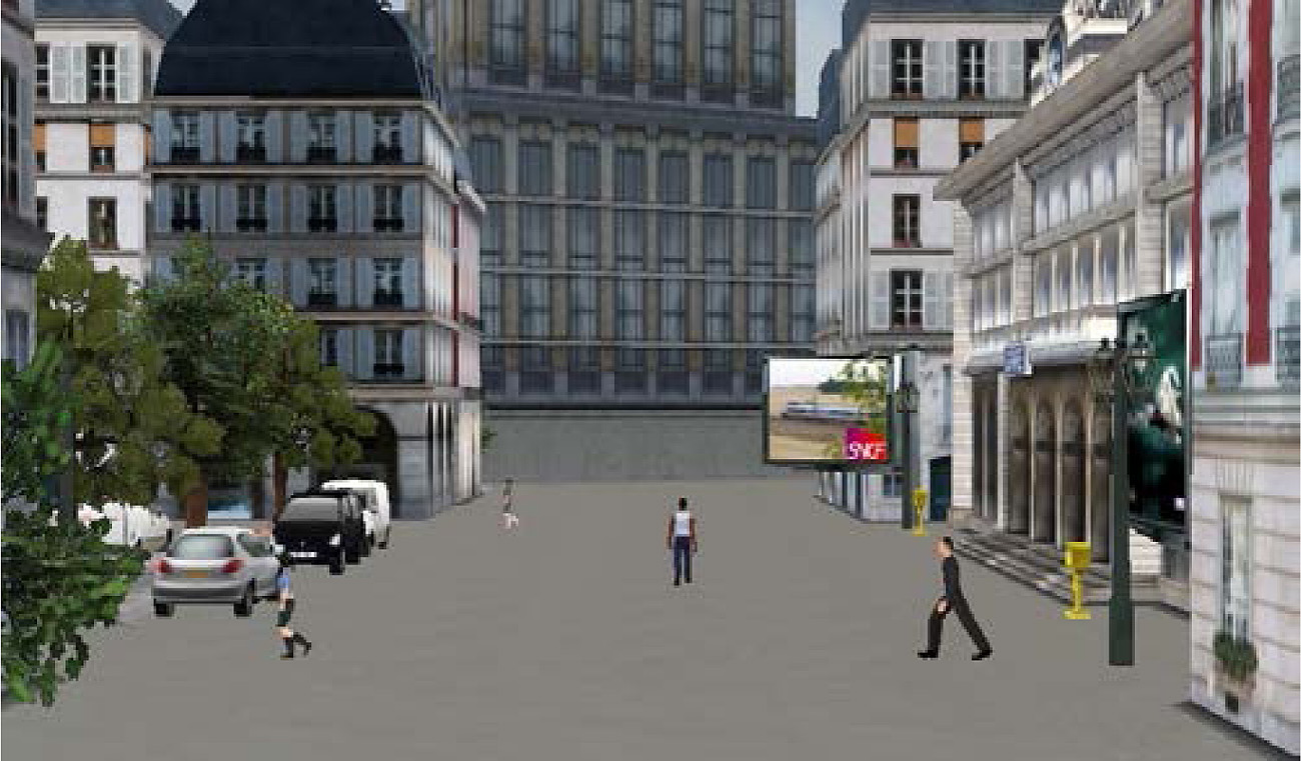
**The virtual town: a view on a pedestrian path with some people walking, buildings, and street furniture**.

**FIGURE 2:**
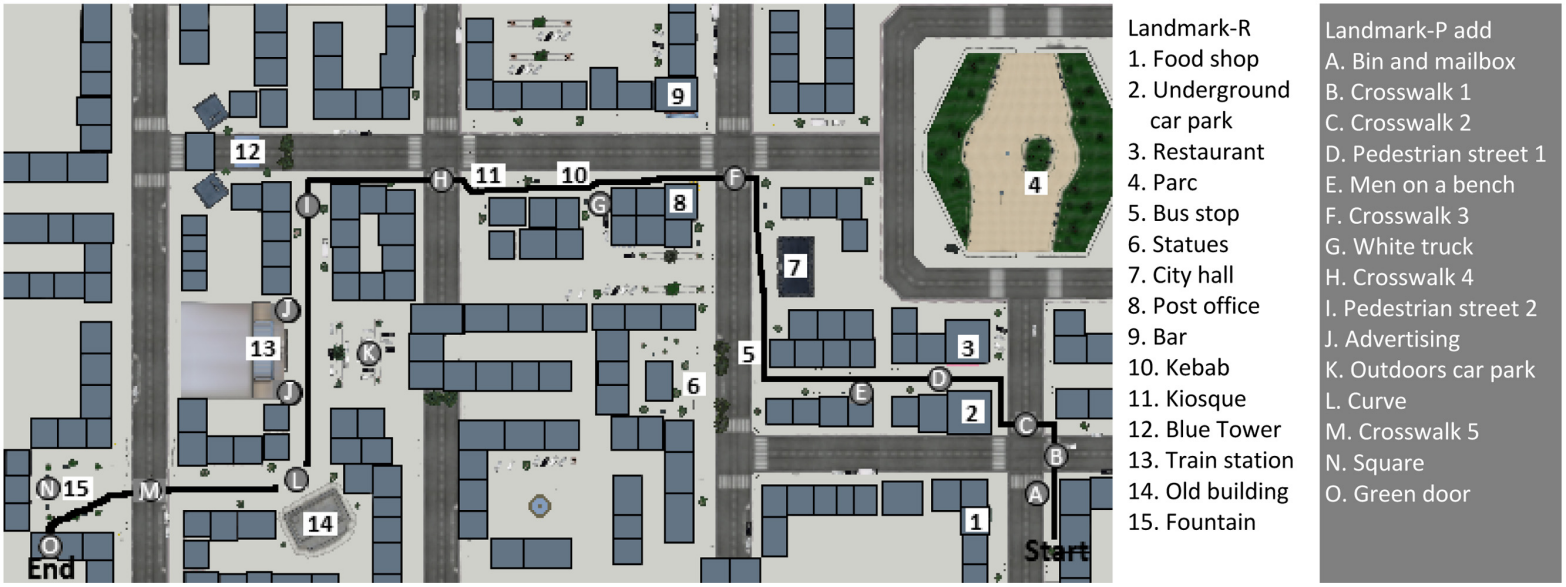
**Map of the environment with the route (in black), the landmarks used in the recognition task (numbers in white), and the landmarks additionally mentioned by the participants in their verbal descriptions (letters in grey)**.

The training and test itineraries were built with the software ‘Virtools 5.0. Dassault system.’ Itineraries were in virtual towns (e.g., **Figure 1**) and contained specific entities (town hall, train station, shops, blue tower, fountain), non-specific buildings (habitation and office buildings), and other non-specific entities (street furniture, people, vehicles, streets, pedestrian paths). The training route was designed with two crossroads and two changes of direction. The test route contained 14 crossroads, seven of which involved a change of direction (**Figure 2**). Participants watched the route as a pedestrian on a 15.4 inches computer screen (Fujistsu Celsius H270).

For the map-drawing task, a map of the town showing the shape of the buildings, the roads, and three landmarks (two relevant, one distractor) was presented on a sheet of paper.

**FIGURE 3:**
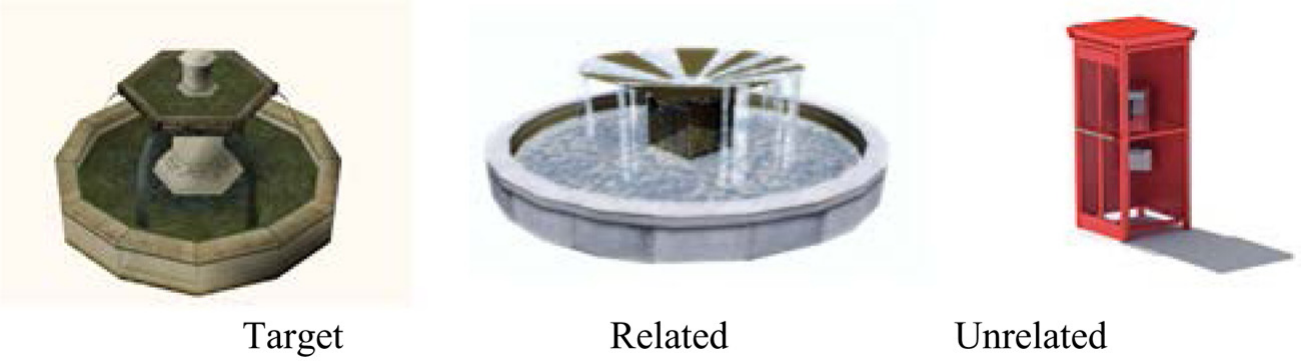
**Example of the three types of pictures used in the visual recognition task: target (the fountain encountered during the route), related distractor (another fountain that was visually different), unrelated distractor (an entity that was verbally and visually different from the target)**.

**Figure 3** illustrates the visual recognition task of landmarks. This task was composed of 34 different pictures: 15 landmarks extracted from the route without context (‘targets’); 15 pictures showing entities that were categorically closely related to the target and had the same name (e.g., “fountain”) but looked different (‘related distractor’); and four pictures showing categorically unrelated entities that were verbally and visually different from the target (‘unrelated distractor’).

**FIGURE 4:**
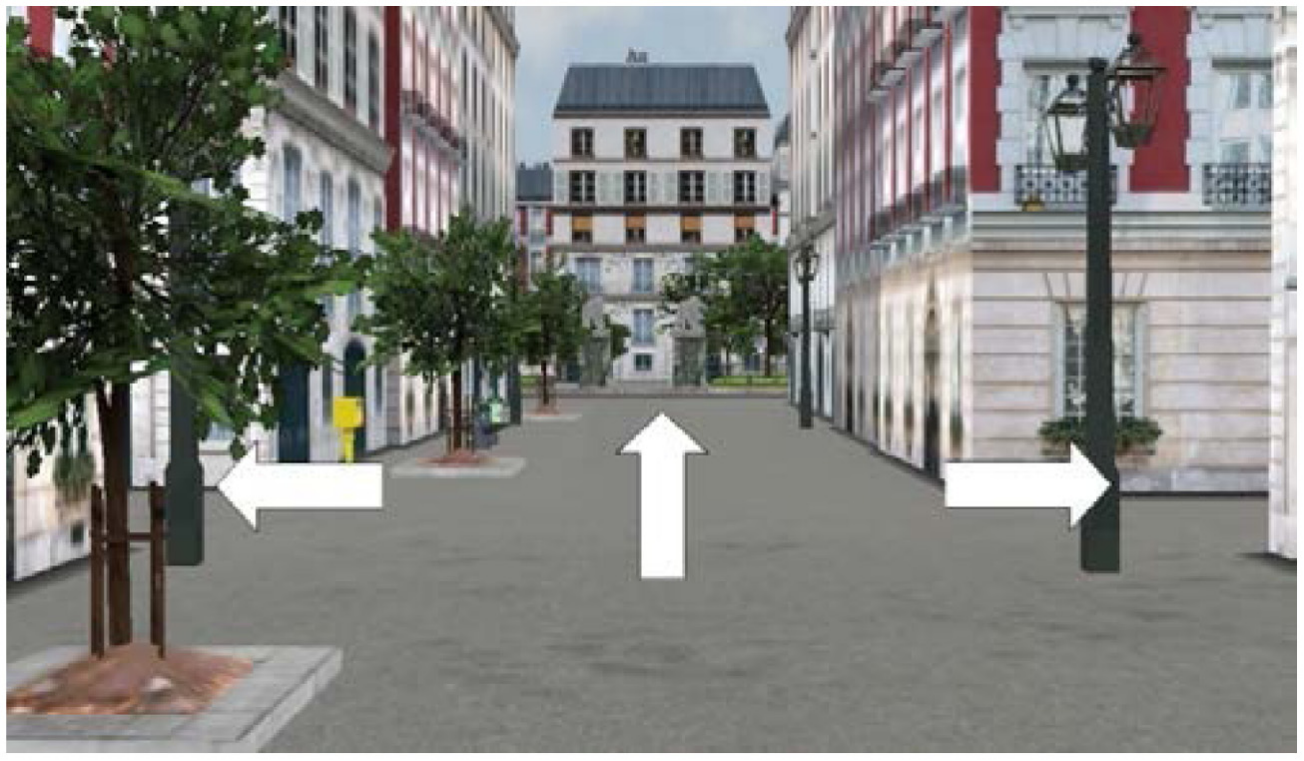
**Example of a picture in the direction-choosing task**.

For the direction-choosing task, two pictures of crossroads extracted from the training route and 11 from the test route were selected (the clearest and most unambiguous ones). On each picture (e.g., **Figure 4**), arrows indicated the possible directions (straight ahead, left, right).

For the map recognition task, the correct route was reproduced on the map and presented with three incorrect itineraries. Four itineraries on the map of the town were presented for the training phase and four different ones were then presented for the test phase.

#### Complementary non-verbal and verbal tasks

In order to measure visuo-spatial (attention, working memory, perception of direction, and mental rotation) and verbal abilities (comprehension and production of spatial lexical items as well as sentence comprehension and storytelling), the following standardized tests were proposed. Tests were selected to be appropriate for all age groups, given that most other available tests only concern particular ages, as well as to be playful and varied. First, with respect to non-verbal abilities, selective attention was measured with the ‘cross test’ from the NEPSY (Korkman et al., 2003), which includes scores for accuracy and response speed. Short-term and working memory abilities were measured with the ‘digit span test’ from the MEM-III (Wechsler, 2001), the ‘Corsi Block’ test from the MEM-III (Wechsler, 2001), and the ‘binding span test’ (Picard et al., 2012). Perception of direction was measured with the ‘arrow task’ from the NEPSY (Korkman et al., 2003). Adult mental rotation ability was measured with the ‘Mental Rotation Test’ (Vandenberg and Kuse, 1978). In addition, the following verbal abilities were tested: sentence comprehension was measured with the ‘C2’ sentence-picture matching sub-test from the ELO test (Khomsi, 2001), involving both simple sentences (e.g., “*the bear is sleeping*”) and more complex ones (e.g., “*the girl [whom the boy hurt] wears glasses*”); the production and comprehension of spatial prepositions (*in, on, under, above, below, left, right, between, in front of, behind*) and of motion verbs most of which indicate path in French (*up, down, into, out of, above*) were measured with the ‘House test’ (Hickmann et al., 2013); and narrative production ability test was evaluated with the ENNI (Schneider et al., 2005), which includes measures for appropriate referent introductions (use of indefinite forms on first mentions) and for story grammar (inclusion and organization of story units, such as setting, initiating event, internal response, internal plan, attempt, outcome, and reaction).

### PROCEDURE

**FIGURE 5:**
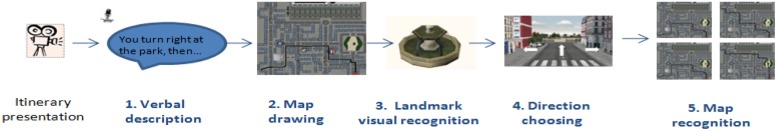
**Order of the route tasks**.

The whole experiment lasted 1h 30 min and was administered in a quiet room by the same experimenter. It was divided into two or three sessions for children at school. Adults performed all tasks in one session at the University Paris Descartes. The experiment comprised two main parts: all tasks concerning the test route (hereafter “route tasks”) were presented first, then all tasks assessing general cognitive (visuo-spatial and verbal) abilities. Part one devoted to the route began with a training phase, followed by a test phase that was composed of five different tasks in a specific order, summarized in **Figure 5**. This order was chosen in such a way as to reduce possible task-order effects with particular attention to minimizing the influence of incoming information presented during the visuo-spatial tasks on performance in verbal production. As an example, mentions of landmarks in verbal descriptions could be influenced by the fact that some entities could be identified as well as named during the map-drawing task. This type of influence was considered to be more damageable than the influence of the verbal task in the reverse order.

#### Learning phase

Participants had to watch on a 15.4 inches computer screen a movie of the route in a virtual town (training or test) and to memorize it. In order to facilitate memorization, the movie was shown twice. Previous pre-testing on 12 adults showed that two presentations were necessary and sufficient for most adults to construct a representation of the environment allowing them to perform all the tasks based on the route (as also in Gras et al., 2013).

#### Verbal description

Participants had to describe the route orally to someone who did not know the town and would have to reproduce the route. Descriptions were recorded then transcribed and coded with the software CLAN in CHILDES (MacWhinney, 2000).

#### Map-drawing

Participants had to draw the route on a map as well as to recall (orally) and place relevant landmarks on it.

#### Visual recognition of landmarks

Participants had to recognize landmarks from the route among several pictures presented on the screen with Eprime software. Each participant pressed a green button when they thought the picture belonged to the route and a red one when it did not. Each participant saw 15 pictures (of the 34 pictures), presented one by one in random order, and each time they heard the name of the picture: seven targets selected from the set of 15, as well as four related and four unrelated distractors. The pictures presented were counterbalanced between participants using four different lists so that each landmark was presented in both the target and related item conditions but that each participant saw only one condition for a same landmark.

#### Choosing directions

On the basis of pictures of crossroads extracted from the route, participants had to choose which direction to take (straight, right, left) by pressing one of the arrows of the keyboard. They saw 11 pictures presented one by one, in the order in which they occurred in the route.

#### Map recognition

Participants had to choose which route was the correct one among four different choices.

In the second part of the experiment dedicated to the assessment of general cognitive and verbal abilities, the attention task (Korkman et al., 2003) was proposed between the training and test phases. Other tasks were presented in a fixed order right after the tasks concerning the route.

## RESULTS

The results are presented in three main sections concerning first the route tasks, then their interrelations, and finally their relationships with visuo-spatial and verbal abilities as measured by independent standardized tests.

### ROUTE TASKS

The results concerning the tasks about the virtual route focus on the production and recognition of landmarks and directions. Performance in (verbal and non-verbal) production was based on how well participants could mention landmarks and directions in their verbal description and in the map-drawing task. Performance in (non-verbal) recognition was based on how well they could visually recognize landmarks, choose directions, and recognize the correct map. For each production or recognition measure, age effects were tested using one-way or repeated ANOVAs. Fisher LSD *post hoc* analyzes specify between-group differences (age groups) and within-group differences (conditions).

With respect to verbal descriptions, a preliminary analysis is necessary concerning participants’ overall productivity since this measure could influence how many landmarks and directions they produce: the more talkative participants are, the more likely they might mention various elements of the route. In order to check this variable, global productivity was measured using the total number of words produced by participants. As expected, a one-way ANOVA with Age (4) as between-subject factor shows that the total number of words uttered increases with age [*F*(3,75) = 11.53, *p* < 0.0001, η^2^= 0.32], particularly between each of the children’s age group and adult age (6 years: *x* = 149.45, SE = 50.36; 8 years: *x* = 2.71.94, SE = 55.67; 10 years: 305.59, SE = 54.19; adults: *x* = 569.45, SE = 52.81; *p* < 0.001), whereas other age differences were not significant (6 vs. 8 years and 8 vs. 10 years).

#### Production of landmarks in verbal descriptions and in the map-drawing task

**FIGURE 6:**
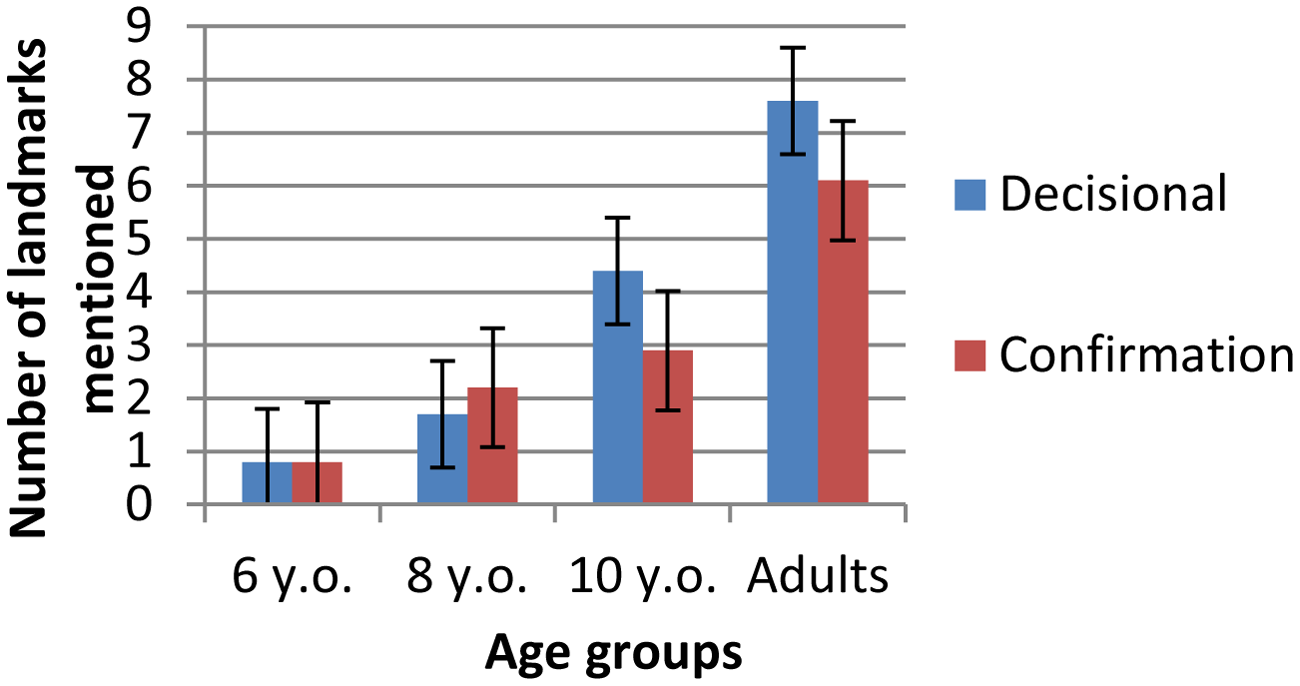
**Mean scores for decisional and confirmation landmarks-V**.

Thirty different elements (specific buildings, entities, streets, or pedestrian paths) were produced as landmarks in the description of the test route. Recall that half of the landmarks in the test route were decisional (at a point where direction changed) and half confirmed the way (non-changing direction). **Figure 6** shows the number of landmarks mentioned (thereafter called landmarks-V) by participants in each age group during their verbal descriptions as a function of whether these denoted entities served as decisional vs. confirmation landmarks-V in the test route.

A repeated 2 × 4 measure ANOVA (position of landmarks-V × age) revealed a significant effect of age [*F*(3,75) = 61.35, *p* < 0.0001, η^2^= 0.71] and of position [*F*(1,75) = 8.52, *p* < 0.01, η^2^= 0.10], as well as an interaction between these two factors [*F*(3,75) = 6.02, *p* < 0.0001, η^2^= 0.19]. Older participants produced more landmarks-V than younger ones and decisional landmarks-V (*x* = 3.60, SE = 0.22) were more frequent overall than confirmation landmarks-V (*x* = 3.01, SE = 0.17). However, *post hoc* analyzes comparing the position of landmark-V within each age showed that 6-year-olds (*p*> 0.05) and 8-year-olds (*p* > 0.05) mentioned as many confirmation and decisional landmarks (for 6 years: *x* = 0.81, SE = 0.42 vs. *x* = 0.77, SE = 0.32 and for 8 years: *x* = 1.67, SE = 0.47 vs. *x* = 2.22, SE = 0.35), whereas 10-year-olds (*p* < 0.001) and adults (*p* < 0.001) mentioned more decisional landmarks-V than confirmation landmarks-V (for 10 years: *x* = 4.37, SE = 0.45 vs. *x* = 2.95, SE = 0.35 and adults: *x* = 7.55, SE = 0.44 vs. 6.10, SE = 0.34). In addition, comparing ages for each landmark-V position showed that decisional landmarks-V were as frequent at ages 6 and 8 but increased significantly between ages 8 and 10 (*p* < 0.0001) as well as between age 8 and adult age (*p* < 0.0001). As for confirmatory landmarks-V, they increased between ages 6 and 8 (*p* < 0.05), did not differ at ages 8 and 10, and increased again between age 10 and adult age (*p* < 0.0001).

**Table 1** summarizes performance with landmarks during the map-drawing and direction-choosing tasks. With respect to the numbers of correct mentions and placements of landmarks on the map (hereafter “landmarks-M”) a one-way ANOVA with Age (4) as a between factor revealed a significant effect of age [*F*(3,75) = 49.56, *p* < 0.0001, η^2^= 0.66] on mentions of landmarks-M. *Post hoc* analyzes showed that participants mentioned increasingly more landmarks-M with age (6 years: *x* = 0.55, SE = 0.41; 8 years: *x* = 1.89, SE = 0.45; 10 years: *x* = 3.55, SE = 0.44; adults: *x* = 7.45, SE = 0.43) and that all age differences were significant (6 vs. 8 years: *p* < 0.05; 8 vs. 10 years: *p* < 0.05; 10 years vs. adults: *p* < 0.0001). A one-way ANOVA with Age (4) as a between factor also revealed a significant effect of age [*F*(3,75) = 37.70, *p* < 0.0001, η^2^= 0.60] on placement of landmarks-M. *Post hoc* analyzes did not show any significant difference between ages 6 (*x* = 0.14, SE = 0.42) and 8 (*x* = 1.00, SE = 0.46) nor between ages 8 and 10, but 10-year-olds placed fewer landmarks-M than adults (*x* = 2.16, SE = 0.45 vs. 6.20, SE = 0.44, *p* < 0.0001).

**Table 1:** Mentions and placements of landmarks during the map-drawing task, and performance in the direction-choosing task.

	6 years old (N = 22) means (SE)	8 years old (N = 18) means (SE)	10 years old (N = 19) means (SE)	Adults (N = 20) means (SE)
Landmarks mentioned during map-drawing	0.55 (0.41)	1.89 (0.45)	3.55 (0.44)	7.45 (0.43)
Landmarks placed on map	0.14 (0.42)	1.00 (0.46)	2.16 (0.45)	6.20 (0.44)
Rate of choosing directions	0.48 (0.03)	0.68 (0.04)	0.74 (0.04)	0.90 (0.04)

#### Visual recognition of landmarks

**FIGURE 7:**
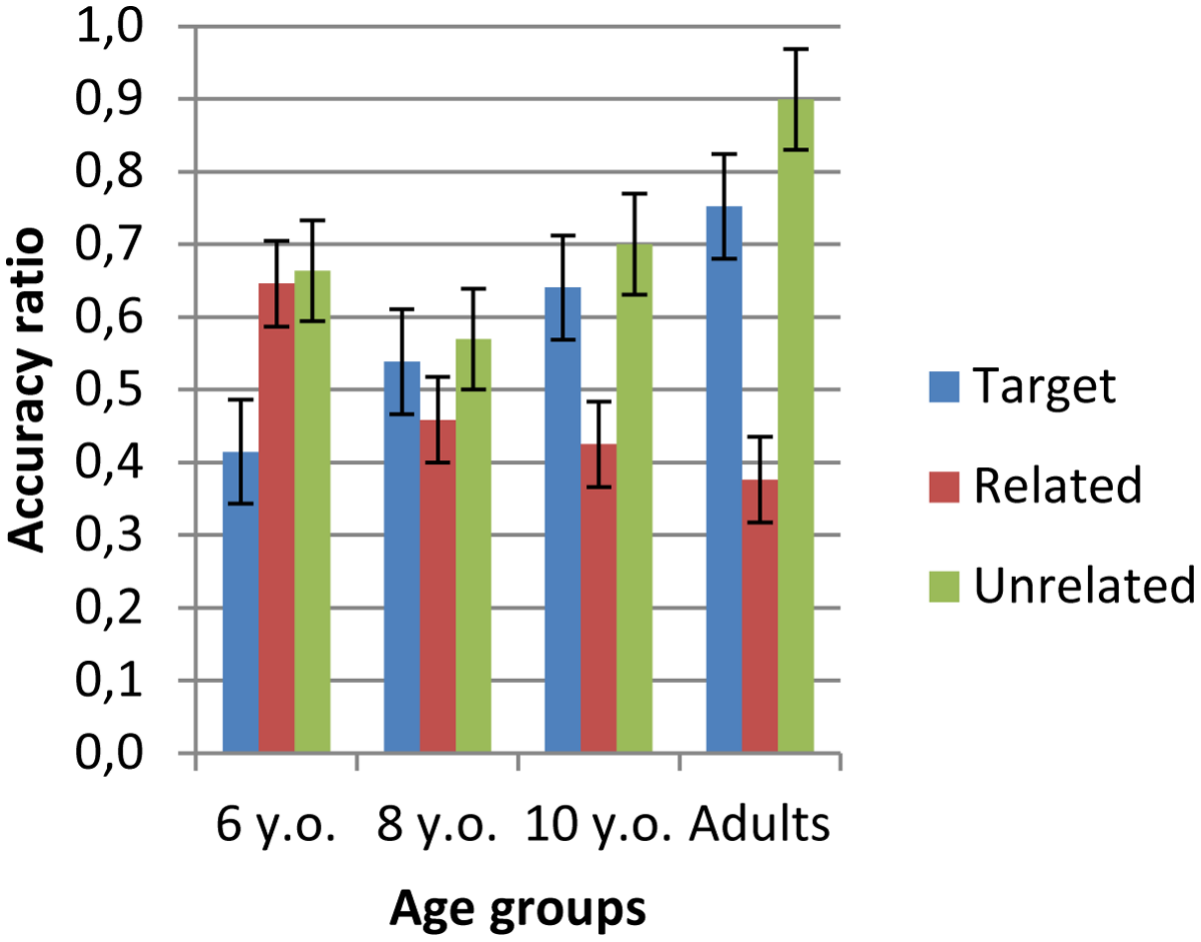
**Accuracy ratio for targets, related and unrelated distractors by age**.

Recall that participants were shown pictures of three types of entities and asked whether they had seen them in the test route: targets (correct response “yes”) and distractors, i.e., a related member of the same category as the target or a totally unrelated entity (correct response “no” in both cases). In order to evaluate participants’ visual recognition of landmarks (hereafter “landmarks-R”), an accuracy ratio was calculated on the basis of the average number of correct responses for each type of picture as a function of the total number of pictures of each type that were presented (seven targets, four related distractors, four unrelated distractors). The results are shown in **Figure 7**.

A repeated measure ANOVA was carried out with Age (4) as a between-subject factor and Picture type (3) as a within-subject factor on landmark-V as the dependent variable. The results revealed an effect of age [*F*(3,75) = 5.11, *p* < 0.01, η^2^= 0.17] and of picture type [*F*(2,150) = 19.36, *p* < 0.0001, η^2^= 0.21] as well as an interaction between these two factors [*F*(6,150) = 7.03, *p* < 0.0001, η^2^= 0.22]. Correct recognition of targets and correct rejects of unrelated distractors increased with age. In contrast, correct rejects of related distractors decreased with age. *Post hoc* analyzes showed different performance profiles at each age. At 6 years correct answers were less frequent for targets (*x* = 0.44, SE = 0.04) than for related distractors (*x* = 0.65, SE = 0.05, *p* < 0.01) and for unrelated distractors (*x* = 0.63, SE = 0.05, *p* < 0.01). At 8 years no significant picture effect was observed (target: *x* = 0.54, SE = 0.05; related distractor: *x* = 0.46, SE = 0.06; unrelated distractor: *x* = 0.06, SE = 0.06). At 10 years correct answers for related distractors (*x* = 0.42, SE = 0.05) were less frequent than for targets (*x* = 0.64, SE = 0.05, *p* < 0.01) and for unrelated distractors (*x* = 0.70, SE = 0.06, *p* < 0.001). Adults’ correct answers were less frequent for related distractors (*x* = 0.38, SE = 0.05) than for targets (*x* = 0.78, SE = 0.04, *p* < 0.01), and the latter were less frequent than those for unrelated distractors (*x* = 0.90, SE = 0.05, *p* < 0.05). Correct answers for related distractors were more frequent at 6 years than at 8 years (*p* < 0.05) but were not significantly different for targets and for unrelated distractors. Correct answers for unrelated distractors were less frequent for 10-year-olds than for adults (*p* < 0.01) but not significantly different for targets and related distractors.

#### Production of directions in verbal descriptions and in the map-drawing task

**FIGURE 8:**
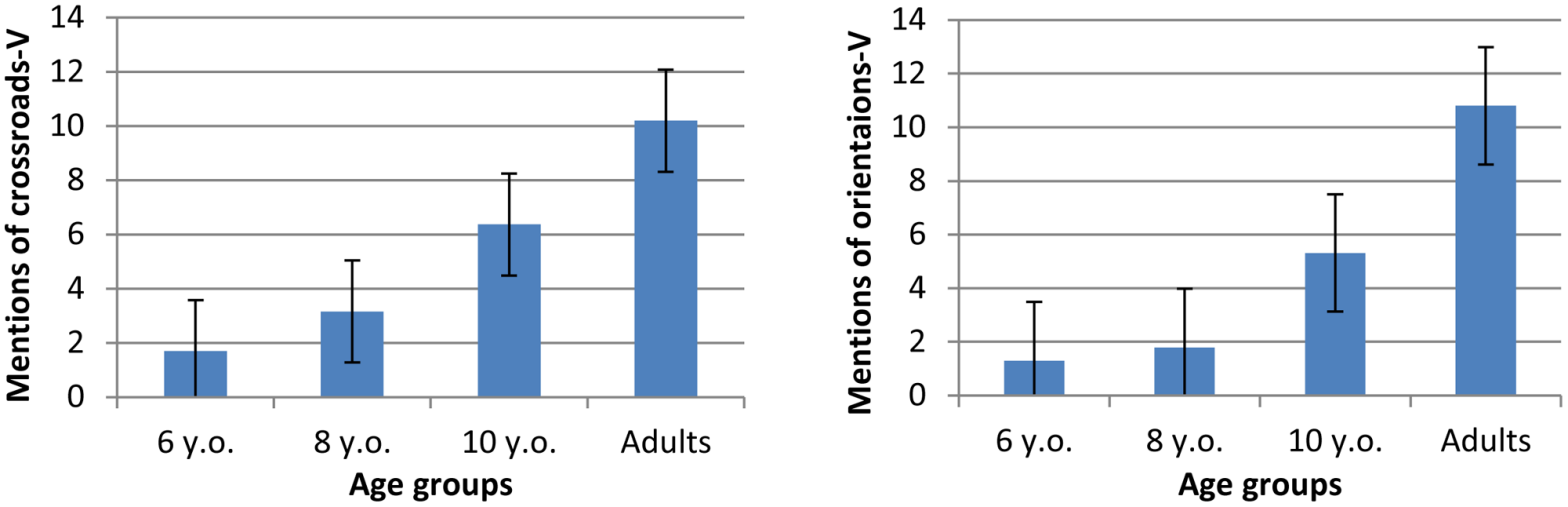
**Mean number of mentions of crossroads and orientations in verbal descriptions**.

For the indication of directions in verbal descriptions (hereafter “directions-V”) and during map-drawing (hereafter “directions-M”), we made a distinction between the indication of a crossroad (hereafter “crossroads-V,” e.g., ‘there is a corner’) and the orientation taken at a crossroad (hereafter “orientations-V”, e.g., ‘left, right, ahead’). The mean number of crossroads-V and of orientations-V mentioned in verbal descriptions was calculated per group (maximum 14). The results are shown in **Figure 8**.

A one-way ANOVA with Age (4) as a between factor on verbal mentions of crossroads-V revealed a significant effect of age [*F*(3,75) = 28.56, *p* < 0.0001, η^2^= 0.53]. *Post hoc* analyzes did not show any significant difference between 6 years (*x* = 1.77, SE = 0.67) and 8 years (*x* = 3.33, SE = 0.74), but increasingly more mentions of crossroads-V from 8 years on (8 vs. 10 years: *p* < 0.01; 10 years vs. adults: *p* < 0.001; with for 10 years: *x* = 6.37, SE = 0.72 and for adults: *x* = 10.20, SE = 0.70). A one-way ANOVA on correct mentions of orientations-V revealed a significant effect of age [*F*(3,71) = 43.07, *p* < 0.0001, η^2^= 0.62]. *Post hoc* analyzes did not show any significant difference between ages 6 (*x* = 1.36, SE = 0.63) and 8 (*x* = 1.89, SE = 0.70), but increasingly more mentions of orientations-V from 8 years on (8 vs. 10 years: *p* < 0.001; 10 years vs. adults: *p* < 0.0001; for 10 years: 5.32, SE = 0.68 and for adults: *x* = 10.80, SE = 0.66).

**FIGURE 9:**
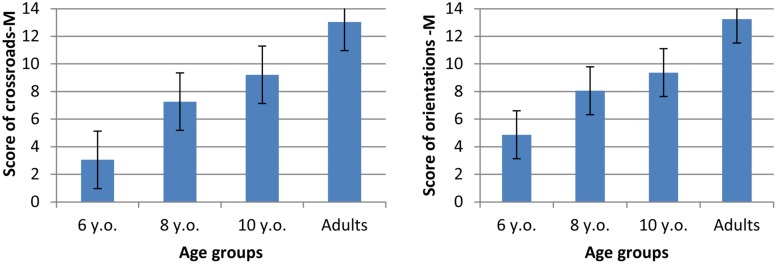
**Mean scores for crossroads-M and orientations-M indicated on the map**.

The indication of directions-M (on the map) was evaluated by counting the number of correct indications of crossroads-M (turn at the correct corner) and orientations-M (ahead, left, right). Correct responses (maximum 14) are presented in **Figure 9**.

A one-way ANOVA with Age (4) as a between factor on indications of crossroads-M revealed a significant effect of age [*F*(3,75) = 47.46, *p* < 0.0001, η^2^= 0.66]. *Post hoc* analyzes showed that participants indicated increasingly more crossroads-M with age (6 years: *x* = 3.05, SE = 0.59; 8 years: *x* = 7.28, SE = 0.65; 10 years: *x* = 9.21, SE = 0.63; adults: *x* = 13.05, SE = 0.62) and that all age differences were significant (6 vs. 8 years: *p* < 0.001; 8 vs. 10 years: *p* < 0.05; 10 years vs. adults: *p* < 0.0001).

One-way ANOVA with Age (4) as a between factor on correct orientation-M also revealed a significant age effect [*F*(3,75) = 35.65, *p* < 0.0001, η^2^= 0.59]. *Post hoc* analyzes showed that correct orientation-M increased with age (6 years: *x* = 5.05, SE = 0.56; 8 years: *x* = 8.06, SE = 0.62; 10 years: *x* = 9.37, SE = 0.60; adults: *x* = 13.30, SE = 0.58) and that all age differences were significant (6 vs. 8 years: *p* < 0.0001; 8 vs. 10 years: *p* < 0.05; 10 years vs. adults: *p* < 0.0001).

#### Recognition of directions

Performance in the direction-choosing task was based on means for accurate visual recognition of directions (hereafter “directions-R”) presented in **Table 1**. A one-way ANOVA with Age (4) as a between factor on directions-R revealed a significant age effect [*F*(3,75) = 24.51, *p* < 0.0001, η^2^= 0.50]. *Post hoc* analyzes showed that participants recognized increasingly more directions-R with age (6 years: *x* = 0.48, SE = 0.03; 8 years: *x* = 0.68, SE = 0.04; 10 years: *x* = 0.74, SE = 0.04; adults: *x* = 0.90, SE = 0.04) and that all age differences were significant (6 vs. 8 years: *p* < 0.001; 8 vs. 10 years: *p* < 0.05; 10 years vs. adults: *p* < 0.01).

### CORRELATIONS AMONG ALL TASKS CONCERNING THE TEST ROUTE

To assess the multidimensional nature of spatial mental models, we examined the relationships among different tasks assessing the same information in different formats (verbal or visuo-spatial). We also paid particular attention to the relationships between performance with landmarks and with directions, as a means of evaluating the dependency between these two forms of spatial knowledge. All measures in the tasks were strongly correlated with age, as expected given the age effects described above. Therefore, in order to examine individual differences, partial correlations controlling for age are presented (**Table 2**).

**Table 2:** Partial correlations (controlled for age) among the different route measures (significant value in bold if up to p < 0.05; with Bonferroni correction).

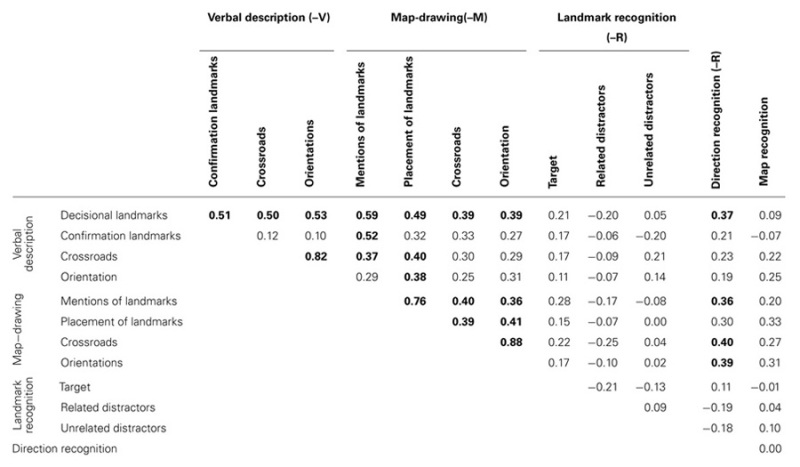

First, with respect to *verbal descriptions*, the following correlations were found: mentions of decisional landmarks-V increase with mentions of confirmation landmark-V, crossroad and orientation in verbal descriptions, with map-drawing (indications of landmarks-M and of directions-M), and with the recognition of directions (directions-R) but not with the recognition of landmarks (landmarks-R), nor with map recognition. In contrast, confirmation landmark-V increased only with mentions of landmarks-M.

Second, with respect to *map-drawing*, the following results were observed: both mentions and placements of landmarks on the map increase with mentions of decisional landmarks-V and with indications of directions-M (crossroads-M and orientation-M). Mentions of landmarks-M and of directions-M (crossroads-M and orientations-M) also increased with directions-R, whereas placement of landmarks-M do not.

Third, with respect to *landmark recognition*, no correlations were found.

Fourth, accurate *direction recognition* increases specifically with mentions of decisional landmarks-V and with performance in most map-drawing tasks (not with landmarks placement-M). It is not correlated with correct landmarks-R or map recognition.

This analysis reveals that only decisional landmarks-V and direction-R are strongly linked with other verbal and visual route tasks. Contrary to expectations, however, we did not observe any privileged links among verbal tasks, nor among visuo-spatial tasks.

### CORRELATIONS BETWEEN COGNITIVE ABILITIES AND SPATIAL KNOWLEDGE

A final set of analyzes aimed to determine whether spatial knowledge (as measured by the route tasks) were related to more general abilities (as measured by independent standardized tests), in order to examine individual differences. Particular attention was placed on whether verbal vs. non-verbal spatial performance was related to general visuo-spatial vs. verbal abilities. For this purpose, we calculated Spearman correlations between each measure of general ability and each measure of spatial knowledge, controlling for age (see **Table 3**). Only significant correlations after Bonferroni’s correction are presented below (*p* < 0.05).

**Table 3:** Partial correlations between cognitive abilities vs performance in the route tasks controlled for age (with Bonferroni’s corrections, significant correlations of up to p < 0.05 are indicated in bold).

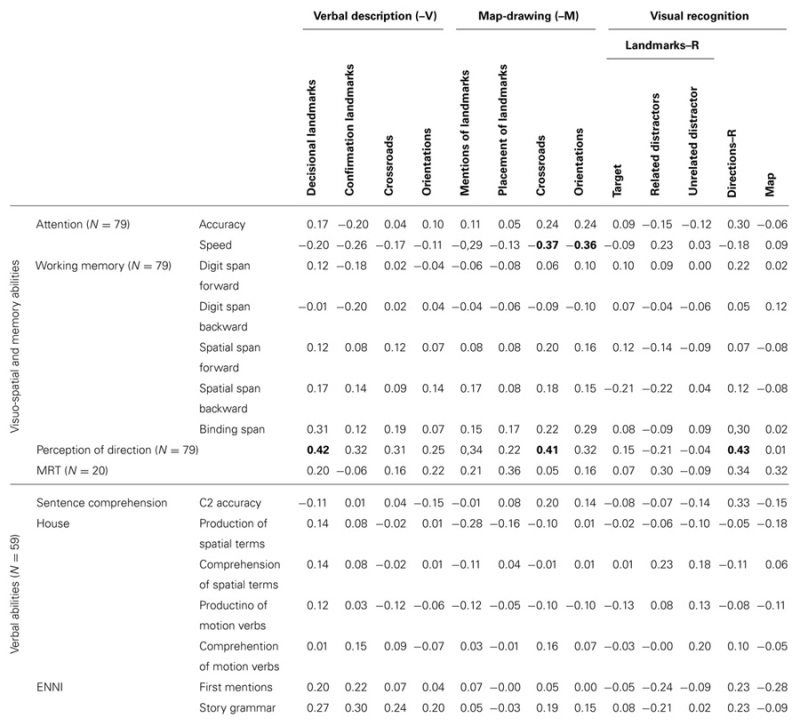

General visuo-spatial abilities measures included the following: visual attention, working memory, perception of directions, and mental rotation. Each of these abilities did not relate in the same way to the measures in the route tasks. Contrary to expectation, attention test accuracy was not much related to route performance. However, with respect to response speed during the attention tasks, the slower the responses, the lower the scores for orientations-M (*r* = -0.36) and crossroads-M (*r* = -0.37). There was no correlation between verbal or visuo-spatial short-term memory, nor between working memory, nor buffer capacities and route tasks. Perception of directions increased with mentions of decisional landmarks-V (*r* = 0.42), of orientations-V (*r* = 0.25) as well as with correct recognition of direction-R (*r* = 0.43). In the adult group, there was no significant correlation between mental rotation and scores in any route tasks.

General verbal abilities were measured only in children. These abilities did not correlate with performance in any of the route tasks.

## DISCUSSION

The general aim of the present research was to inform verbal and non-verbal mechanisms underlying the development of children’s ability to construct spatial representations using knowledge about landmarks and directions. On the basis of a virtual town, we examined the performance of children aged 6, 8, 10 years and of adults in production and recognition tasks using both visual and verbal modalities. The study aimed to address two more specific questions. First, we wanted to assess the development of spatial knowledge with particular attention to the development and role of landmark knowledge in a virtual route. Second, we wanted to examine some of the relations between spatial knowledge and general verbal and non-verbal abilities. The following hypotheses were therefore tested. First, it was expected that knowledge of landmarks should increase with age, as reflected by an increase in the number and quality of the landmarks mentioned and recognized, followed by the later development of route knowledge, as reflected by knowledge of crossroads and orientations in relation to landmark position. As a result, only older children should be able to organize and bind together various components of the route into a global representation. Concerning our second research question, it was expected that the development of spatial representations should be related to increasing general verbal and non-verbal abilities. Attention, which is considered to be a prerequisite for the construction of any representation, was expected to be related to all measures in the route tasks. Other non-verbal (visuo-spatial) abilities were expected to be strongly linked to visual measures of route test, whereas verbal abilities were expected to be mostly linked to verbal measures of spatial knowledge as well as to the ability to bind and to structure all types of information into a coherent representation.

### LANDMARK KNOWLEDGE

As expected, we observed an increase in the number and quality of the landmarks mentioned and recognized between age 6 and adulthood. This increase of performance was observed in both verbal and visuo-spatial tasks. Although the type of information assessed by different modalities in these two types of tasks is similar, the processes involved are different. On the one hand, our analyzes show that increasing mentions of landmarks in verbal descriptions correlate with the number of landmarks correctly mentioned and decisional landmarks placed in the map tasks. Accuracy in landmark placement increased between 6 and 10 years, as also shown in previous studies (e.g., Herman, 1980; Sandamas and Foreman, 2007). On the other hand, it is more difficult for all participants to mention these landmarks in relation to a map than to do so in a verbal description, even though the verbal description task occurred first which should have facilitated subsequent mentions on the map. Most of the children in our study were able to represent spatial relations, as shown for example by their correct production and comprehension of spatial prepositions in the ‘house test.’ One possible explanation for their difficulty in mentioning and placing landmarks in the map task could be a more general difficulty in understanding maps. However, Peter et al. (2010) showed that children as young as 5 and 6 years grasped such symbolic representations as well as spatial terms. It follows that most of the youngest children from our study should have been able to construct a global town representation and to understand the map as a symbolic representation. Another explanation for the difference in performance in verbal and visuo-spatial assessments is that it is more difficult to encode and recall the location of a landmark than the landmark itself. Indicating locations requires remembering a main element and its relationship to some others (e.g., position in relation to another entity, distance, directions ...). Bauer et al. (2012) studied the development of children’s memory for ‘events,’ ‘locations,’ and ‘events with locations’ between 4 and 8 years. A main effect of age was observed for all measures. However, it was only at age 8 that children’s recall of events and of their locations was tightly linked (above chance recognition and positive correlation). This result indicates that the development of long-term memory for the location of events is difficult and emerges late. If we assume that landmark placement is equivalent to remembering events in their location, then children’s difficulty in binding events and their location might explain their difficulty in recalling and placing landmarks from the route in the map tasks of our study. Indeed, it is possible to view the knowledge that underlies landmark placement as being equivalent to that involved in remembering events in their location.

### FROM LANDMARK TO ROUTE KNOWLEDGE

In our study route knowledge is reflected in the ability to mention crossroads and orientations, as well as to bind all types of information with landmarks in verbal descriptions and to indicate them on maps. Our results show that mentioning directions in a verbal description or indicating directions on a map is very difficult for children at 6 and 8 years. Knowledge of directions starts developing between 8 and 10 years and continues to evolve until adulthood (where most participants mention all correct crossroads and orientations). This knowledge is also correlated with map recognition – which corresponds to a global view of the route, as well as with the ability to indicate the position of the landmarks mentioned. As in Cohen and Schuepfer (1980), who used slide presentation, or in Jansen-Osmann and Wiedenbauer (2004), who used virtual navigation in a maze with or without landmarks, we found that landmarks that were adjacent to correct turns (i.e., decisional landmarks) were mentioned more often than those that were not adjacent to changing points (i.e., confirmation landmarks). In the two previous studies this difference in performance with these two types of landmarks (decisional > confirmation) was observed in all age groups (from 7 years to adulthood). In our study 10-year-old children and adults mentioned decisional landmarks more frequently than confirmation ones, whereas younger children mentioned the two types as frequently. Decisional landmarks are most important in order to reproduce the route in navigation with minimal information (Michon and Denis, 2001). Their specific role is confirmed by the fact that the frequency with which they are mentioned in a verbal description correlates with the mentions and recognition of directions (crossroads and orientations), whereas this relation is not observed for confirmation landmarks.

### DOES LANDMARK KNOWLEDGE FACILITATE THE DEVELOPMENT OF ROUTE KNOWLEDGE?

Taking into account landmark position (which involves more decisional landmarks mentioned and used than confirmation landmarks) seems difficult for children as shown by the non-significant differences between mentions of decisional and confirmation landmarks in the younger children. It appears late in development and increases with the ability to use, indicate, and bind multiple types of information. Contrary to Jansen-Osmann and Wiedenbauer (2004), but consistent with Cohen and Schuepfer (1980), the performance of older children in our study is lower than that of adults. Thus, the absence of significant differences in performance between older children and adults that was previously attributed to the use of virtual reality was not observed in our study, possibly because our world was more complex than the maze presented in the other studies. Jansen-Osmann et al. (2007) show that the more complex a town is, the more difficult it is to represent its configuration. Virtual reality could indeed help enhance performance in children (Jansen-Osmann and Wiedenbauer, 2004) but only if complexity is sufficiently low. This difference could also be explained by the conditions under which participants were asked to memorize the route, given that active and passive encoding have been shown to affect memory differently (Chrastil and Warren, 2012). The participants in Jansen-Osmann and Wiedenbauer’s (2004) study actively navigated in the virtual maze, whereas our participants passively watched a movie.

The developmental change observed during the acquisition of landmark and route knowledge is greater between 6 and 8 years than between 10 years and adulthood. This result indicates a gradual development of spatial knowledge that is consistent with earlier hypotheses (Piaget and Inhelder, 1948; Siegel and White, 1975). The late acquisition of route knowledge can be related to children’s difficulties in organizing and ordering multiple types of information, including detailed knowledge of landmarks, then knowledge of routes, that are necessary to develop a general and complex representation of the route. Route knowledge is complex since it involves both landmark and direction knowledge. However, it is also possible that the gradual development observed in our study might be task-dependent, therefore requiring further research with other tasks and stimuli. For example, younger participants rarely mention landmarks or directions, but their performance seems to improve with recognition tests.

### VERBAL AND VISUO-SPATIAL ENCODING OF INFORMATION IN THE SPATIAL MODEL

Interestingly, this increase in large-scale representation is observed in both verbal and visuo-spatial assessments, which show correlations in performance. Mentions of landmarks increase in number and in quality, while at the same time visual recognition abilities also increase and seem to be biased by verbal factors. In particular, participants’ correct recognition of landmarks improved with increasing age while their false recognition of unrelated distractors decreased. At the same time, false memory of related distractors increases (i.e., entities that belonged to the same category and had the same name as targets but were visually different), perhaps reflecting a semantic bias. Thus, although increasing landmark recognition may result from an increase of memory capacity during development, participants’ increasing false recognition of related distractors suggests that they do not merely use the visual information provided by the pictures. Rather, they may also rely on a ‘concept’ that includes a number of entity properties and can be expressed in a word that facilitates encoding, e.g., the function of all entities we call “fountains” is to provide water so that any object that provides water and that has related features such as a certain shape may be recognized as the landmark encountered on the route. Although this result suggests that lexical knowledge may influence the construction of representations during the route tasks, it contrasts with the findings of Hund and Plumert (2003). Recall that these authors consider an object placement task where objects are allocated randomly or by semantic category, and show a decreasing effect with age of the semantic categorization instead of the increasing effect observed in our study. The decrease of the semantic bias that was observed by Hund and Plumert (2003) can be attributed to children’s increasing accuracy in differentiating entities from the same category. It could reflect the fact that they remember the visual characteristics of landmarks as long as they are visually distinct, thus freeing memory load. In our study, the increasing bias we observed may reflect adults’ reliance on verbal labels or on a general concept the development of which is influenced by the acquisition of multiple verbal and visuo-spatial information. The fact that language may have played a role in explaining our recognition results is all the more likely that the procedure in our study might have encouraged a semantic bias. Recall that participants heard the names of the pictures at the same time as they saw the pictures during the recognition task. It is therefore likely that they made use of these verbal labels when constructing their representations. Future research using a different procedure is clearly necessary in order to determine the relative role of language in this process. In particular, it would be interesting to see if the same result emerges when lexical information is not provided during the visual presentation of the stimuli.

### SPATIAL KNOWLEDGE AND INDIVIDUAL DIFFERENCES IN GENERAL ABILITIES

Finally, given Purser et al.’s (2012) results, we hypothesized that quantitative and qualitative developmental change in large-scale representations should be supported by both verbal and non-verbal abilities. Visual abilities were expected to correlate with landmark and route knowledge, especially for visual recognition and for map-drawing. In contrast, verbal abilities were expected to mainly correlate with performance in verbal descriptions of the route as well as with the ability to bind and organize information. In our study, participants had to remember a long and complex route that included many crossroads, buildings, and street furniture encountered in a specific order during navigation. Contrary to expectation and previous studies, only perception of direction and attention speed were associated with increasing abilities to mention and recognize landmarks and directions, whereas working memory or mental rotation were not. It has already been pointed out that attention is a crucial factor in spatial learning involving computer environments (Wilson and Péruch, 2002) but contrary to expectation attention was not correlated to all types of spatial knowledge measured by our tasks. This result as well as the absence of correlation with memory could be explained by the fact that the route had been presented twice. This double presentation should have facilitated the memorization. Purser et al. (2012) explain their results by the development of executive functions, which were not specifically measured in our study and could be an important set of abilities in the development of spatial knowledge construction to be considered in future studies. Our study further shows that small-scale perception of directions, as measured by the arrow test (NEPSY), is important for the construction of spatial knowledge. This perceptual test requires the ability to judge directions, distances and angles, but also to represent an imaginary line. Such abilities are also required for large-scale pointing, often used to measure wayfinding capacities (e.g., Neidhardt and Popp, 2010). Quaiser-Pohl et al. (2004) studied the relationship between spatial abilities and spatial representation of a large-scale environment in second-, fourth-, and sixth- graders. Contrary to our results, no correlation was observed between scores for spatial abilities and for large-scale mapping. The difference between their results and ours could be explained by the fact that different types of measures were used to assess small-scale visuo-spatial abilities. Whereas many studies use visual reasoning and mental rotation tests to measure visual abilities, our tasks measure attention, working memory, and perception of directions. Another explanation lies in the different measures for knowledge of large-scale environments used across studies. Quaiser-Pohl et al. (2004) used a familiar environment, whereas our study involved an entirely new environment that required learning new information in order to construct a spatial representation. This suggests that the perception of direction reflects a process also involved in spatial learning which is more requested than attention, working memory, or mental rotation and therefore should be further explored.

Contrary to expectation, verbal abilities show no relation to performance in the verbal description task or in other tasks measuring knowledge of the environment. The absence of relationships between some verbal skills and spatial representations can be due to our measures. Indeed, a ceiling effect was observed already at 6 years for knowledge of spatial prepositions and of motions verbs. The fact that spatial knowledge was related to non-verbal abilities and not to verbal ones is consistent with Fenner et al.’s (2000) results. The authors asked children to walk in a campus and measured their verbal and visuo-spatial abilities. They found that visuo-spatial abilities were strongly related to wayfinding but not to verbal capacities. However, these results do not exclude the possibility that verbal abilities could play a role in how we construct full-fledged spatial representations and that they interact with visual knowledge of spatial information. Future research testing this hypothesis will require using other verbal tests, for example tests measuring more complex verbal knowledge that show late developments beyond 6 years of age.

To conclude, this experiment studied the development of children’s spatial representation of an urban-like environment. Its originality lies in the use of a complex virtual town with a number of tasks assessing both landmark and route knowledge about this environment as expressed by participants in both verbal and non-verbal (visuo-spatial) modalities. In addition, these tasks about the virtual environment were coupled with independent tests measuring individual differences in this knowledge (verbal and non-verbal abilities). To our knowledge, no other study has included such a large range of measures in order to address several interrelated questions about the development of spatial representations. Results show that the ability to construct complex spatial representations emerges before 6 years, but takes some time to become fully functional. On the basis of children’s performance in our tasks, it seems that they first acquire general knowledge of landmarks, then this knowledge becomes more accurate with age and landmarks are more likely to be selected according to their functional role in the route (decisional vs. confirmation). During the development of landmark knowledge, route knowledge also increases as shown by an increasing knowledge of directions, and it is related to the ability to determine and to make use of decisional landmarks. In this respect, the study replicates some of the results already available in the literature while at the same time contributing to our understanding of the development of spatial knowledge, such as the role of decisional landmarks and knowledge of directions as well as their relation to specific cognitive abilities, and the role of labels in landmark knowledge. The ability to select landmarks must be more fully explored, with particular attention to the verbal and visuo-spatial encoding of landmark and route knowledge. Examining the performance of younger children with complex itineraries would also be interesting in order to determine the precise role of linguistic factors in these developments.

## Conflict of Interest Statement

The authors declare that the research was conducted in the absence of any commercial or financial relationships that could be construed as a potential conflict of interest.
